# The British Journals: Medical Statistics

**Published:** 1839-01

**Authors:** 


### MEDICAL STATISTICS.

### *On the Mortality and Frequency of Disease amongst Children.*

By PETER HENNIS GREEN, M.B.

[THIS is another of a series of communications on the nature and statistics of Children's Diseases, which have appeared from time to time in the same journal and which do infinite credit to their author. We call the attention of the profession to them, having only room at present for the first Table in the present paper, with the accompanying remarks.]

"This table includes the whole of the entries into the Children's Hospital, Paris, during the years 1833-5 inclusive, and embraces a sum total of 9429 cases. In order to show the manner in which 'mortality' is influenced by 'age,' the patients have been divided into four classes, the interval between each being four years.

| AGE | ENTRIES. | | DEATHS. | |
|---|---|---|---|---|
| | Boys. | Girls. | Boys. | Girls. |
| 2 to 4 | 808 | 678 | 334 | 290 |
| 4 to 6 | 519 | 590 | 163 | 170 |
| 6 to 10 | 1407 | 1427 | 209 | 225 |
| 10 to 15 | 2246 | 1754 | 157 | 176 |
| Total...... | 4980 | 4449 | 863 | 861 |

From the above table it appears that the general mortality was 12·98 per cent.; that the mortality amongst the boys was 11·30 per cent.; and that the mortality amongst the girls was 14·85 per cent. Hence disease is more fatal in female than in male children, in the proportion of 14·85 to 11·30.

Let us now see how far the mortality was influenced by the ages of the patients attacked. In the first period (from two to four years) the mortality was 41·99 per cent.; in the second period it had fallen to 27·54 per cent.; while in the fourth period (from ten to fifteen years) it did not exceed 8·32 per cent. Thus we perceive that the mortality is five times greater at the early period of infancy than it is during the last five years of childhood. *Lancet. October* 13, 1838.

---

*Meteorological Results of ten Years' Observations in the Gardens of the Horticultural Society at Chiswick.*

[The publication from which we extract the following table (*The Medical Annual, edited by* Mr. Farr,) is, as usual, distinguished by the fulness, accuracy, and importance of its contents. We the less regret our inability (from want of room) to make further selections from its pages, as we doubt not that it will soon be in the possession of most of our readers. Besides a vast variety of brief statistical articles, interesting to all classes of the profession, the present Number contains several communications of a higher stamp; among which stands conspicuous the admirable "History of the Medical Profession in England," by the learned editor.]

| Thermom. 1826-35. | Mean. | Max. | Min. | Mean Range. | Max. | Min. | Max. in Sun's Rays. | Min. on the Earth. | Mean pressure in inches | Rain Inches. |
|---|---|---|---|---|---|---|---|---|---|---|
| January... | 36°·4 | 41°·4 | 31°·6 | 9°·8 | 60° | 10° | 70° | 3° | 29·965 | 1·284 |
| February.. | 40 ·7 | 46 ·8 | 34 ·6 | 12 ·2 | 65 | 10 | 80 | 4 | 29·935 | 1·530 |
| March..... | 43 ·5 | 51 ·1 | 35 ·9 | 15 ·1 | 75 | 21 | 95 | 8 | 29·955 | 1·410 |
| April....... | 48 ·8 | 58 ·2 | 39 ·4 | 18 ·8 | 78 | 24 | 105 | 12 | 29·890 | 1·870 |
| May ....... | 55 ·7 | 66 ·7 | 44 ·7 | 21 ·0 | 86 | 28 | 126 | 16 | 30·047 | 1·864 |
| June....... | 60 ·8 | 71 ·7 | 49 ·9 | 21 ·8 | 91 | 37 | 125 | 26 | 29·962 | 1·864 |
| July........ | 64 ·0 | 75 ·3 | 52 ·7 | 22 ·5 | 94 | 40 | 126 | 32 | 30·001 | 2·617 |
| August.... | 62 ·3 | 73 ·4 | 51 ·2 | 22 ·1 | 86 | 36 | 120 | 29 | 29·935 | 2·518 |
| September | 57 ·6 | 67 ·8 | 47 ·4 | 20 ·4 | 82 | 32 | 114 | 27 | 29·938 | 2·932 |
| October... | 51 ·8 | 60 ·4 | 43 ·1 | 17 ·3 | 80 | 28 | 97 | 20 | 29·994 | 2·369 |
| November | 43 ·7 | 50 ·1 | 37 ·4 | 12 ·7 | 63 | 20 | 79 | 11 | 29·950 | 1·969 |
| December | 40 ·7 | 45 ·8 | 35 ·6 | 10 ·2 | 58 | 10 | 69 | 6 | 29·993 | 1·789 |
| Year....... | 50 ·5 | 59 ·1 | 42 ·0 | 17 ·1 | 94 | 10 | 126 | 3 | 29·9581 | 240·13 |

